# Sparse Communication for Policy Shaping in Multi-Agent Reinforcement Learning

**DOI:** 10.3390/s26113413

**Published:** 2026-05-28

**Authors:** Jiahao Li, Renjie Li, Nan Wang

**Affiliations:** Department of Electronic Engineering, Faculty of Information Science and Engineering, Ocean University of China, Qingdao 266000, China; jiahaoli@stu.ouc.edu.cn (J.L.); lirenjie371311@stu.ouc.edu.cn (R.L.)

**Keywords:** multi-agent reinforcement learning, communication efficiency, sparse communication, threshold-gated mechanism

## Abstract

Efficient coordination under limited communication is a central challenge in multi-agent reinforcement learning (MARL). Existing approaches often focus on message exchange without explicitly modeling how communication affects policy learning, leading to redundant interactions and limited coordination gains. In this paper, we propose a threshold-gated sparse communication framework built upon QMIX, a monotonic value-decomposition method that mixes individual agent action values into a global team action value. In the proposed framework, communication is integrated into the agent utility function to directly influence policy learning. Each agent encodes local observations into structured representations and activates communication through a learned trigger mechanism. Messages are aggregated via neighbor-constrained attention and incorporated into utility estimation for decentralized decision-making. Experimental results on the StarCraft Multi-Agent Challenge (SMAC) benchmark show that the proposed method improves coordination quality and training stability while significantly reducing communication frequency. On MMM, the Marine–Marauder–Medivac heterogeneous scenario, the communication rate is reduced to approximately 30–38% while achieving up to 96.6% win rate, compared to 92.1% for QMIX. On 10m_vs_11m, a homogeneous scenario where ten allied Marines fight against eleven enemy Marines, communication remains within 28–37% while reaching 88.4% win rate, compared to 85.6% for QMIX. Moreover, on the same task, varying communication thresholds induce clearly differentiated policy behaviors, indicating that sparse communication not only reduces overhead but also plays a critical role in shaping coordination policies. These results demonstrate that selective communication enables efficient coordination while explicitly regulating policy formation.

## 1. Introduction

Multi-agent reinforcement learning (MARL) has become a key framework for solving cooperative decision-making problems in complex environments, with applications ranging from multi-robot systems [1,2] and autonomous driving to large-scale resource scheduling and distributed control [3,4]. In these settings, agents must achieve effective coordination under partial observability and decentralized execution constraints, while training can leverage global information under the centralized training with decentralized execution (CTDE) paradigm [5,6].

In addition to local decision-making, communication is often introduced to alleviate partial observability by enabling agents to exchange complementary information. Early studies demonstrated that learnable communication can significantly improve cooperation in complex tasks [7,8]. Subsequent works have further explored methods to make communication more selective and efficient, for example by learning when to communicate or whom to communicate with [9,10,11]. These advances suggest that communication plays an important role in enabling coordinated behaviors in MARL.

Despite these developments, learning effective coordination remains challenging. Value decomposition methods such as Value-Decomposition Networks (VDN) and QMIX have shown strong performance and training stability in cooperative MARL [5,6,12]. VDN decomposes the team value into a sum of individual utilities, whereas QMIX uses a monotonic mixing network to combine individual action values into a global team action value. However, these approaches do not explicitly model inter-agent information exchange during execution. As a result, agents rely primarily on local observations, which may limit their ability to form structured cooperative behaviors such as coordinated targeting, spatial formation, and fine-grained micro-control, especially in complex environments such as the StarCraft Multi-Agent Challenge (SMAC) [13,14].

A natural direction is to incorporate sparse communication to improve efficiency. Existing studies on sparse communication primarily focus on reducing communication frequency or bandwidth consumption, often by learning gating or scheduling mechanisms [11,15,16]. While these approaches successfully reduce communication overhead, they typically treat communication as an auxiliary information channel and do not explicitly analyze how communication influences the resulting policy. As a consequence, communication may become less frequent but still fails to induce meaningful changes in coordination patterns or policy structure. Recent works also highlight that effective communication should be adaptive and task-relevant, rather than merely sparse [17]. However, how communication affects the emergence of coordinated behaviors remains underexplored.

In this paper, we propose a threshold-gated sparse communication framework built upon QMIX, which explicitly leverages communication to shape policy behavior rather than merely transmitting information. Each agent encodes local observations using a lightweight convolutional neural network (CNN)-based spatial representation and activates communication based on structured deviations in local states. The transmitted messages are aggregated under a neighbor-constrained attention mechanism and integrated into the agent utility network, enabling communication-aware decision-making.

We evaluate the proposed approach on the StarCraft Multi-Agent Challenge (SMAC). The experimental results show that the proposed method improves coordination quality and stabilizes learning while substantially reducing communication frequency. More importantly, the results indicate that sparse and selective communication leads to more structured cooperative behaviors, including more consistent focus-fire patterns and improved micro-control decisions. These findings suggest that communication can play an active role in shaping cooperative strategies, rather than merely serving as a channel for information exchange.

Based on this motivation, we formulate and investigate three verifiable research questions concerning communication efficiency, behavior-level policy changes, and the role of communication in utility-based cooperative policy learning. By doing so, the proposed framework is connected to testable hypotheses, rather than being presented only as an architectural modification to existing value-decomposition methods.

Our main contributions are summarized as follows:

(1) We propose a threshold-gated sparse communication mechanism that enables agents to selectively communicate based on structured changes in local observations. Unlike dense communication baselines that broadcast messages at every decision step, the proposed trigger suppresses redundant transmissions and activates communication only when the learned local state deviation indicates potential coordination demand.
(2) We introduce a communication-aware QMIX framework that explicitly links communication to policy formation. Specifically, activated neighbor messages are aggregated through a neighborhood-constrained attention module and fused into the recurrent individual utility-estimation pathway, so communication context can influence decentralized action selection and centralized value mixing rather than serving as an auxiliary message channel.
(3) We demonstrate that sparse communication can improve coordination and policy structure while maintaining competitive performance on SMAC benchmarks. Beyond win rate and communication ratio, we further analyze behavior-level coordination indicators such as focus-fire consistency, target distribution entropy, spatial separation, and attack–move transitions, showing how threshold-controlled communication affects learned cooperative behaviors.

## 2. Related Works

### 2.1. MARL for Collaboration (Value-Based CTDE)

Multi-Agent Reinforcement Learning (MARL) has become a prominent paradigm for learning cooperative behaviors in multi-agent teams, enabling emergent coordination such as focus-fire, formation control, and synchronized maneuvers in complex partially observable environments [13,14,18,19]. A widely adopted training paradigm is centralized training with decentralized execution (CTDE), where additional global information is used during training to improve stability and credit assignment, while each agent executes using only local information at test time [3,5,6]. Within CTDE, value decomposition methods are particularly effective for cooperative tasks. VDN factorizes the team action value as a sum of individual utilities, providing a simple yet scalable mechanism for credit assignment [6]. QMIX further generalizes value decomposition by learning a mixing network that combines individual action values into a joint $Q_{\mathrm{tot}}$ under a monotonicity constraint, enabling decentralized greedy action selection while optimizing for the team return [5]. Subsequent value-based approaches improve representation learning, temporal credit assignment, and robustness under partial observability, leading to strong performance on standard cooperative benchmarks [12,13,20,21,22]. Despite this progress, cooperative MARL remains challenged by partial observability, non-stationarity induced by simultaneously learning agents, and the need for effective coordination mechanisms beyond implicit coupling through shared rewards [13,23,24]. In particular, when local observations are insufficient to infer teammates’ intents or to align on shared tactical objectives, explicit inter-agent communication becomes a key ingredient for reliable group strategy emergence [7,9,13].

### 2.2. Communication in MARL

To facilitate coordination beyond what value decomposition alone can provide, a line of work studies differentiable communication mechanisms that allow agents to exchange messages end-to-end with the task objective [7,8,17]. Early approaches learn continuous or discrete messages and backpropagate gradients through the communication channel, enabling agents to share hidden representations that encode intent or local context [8]. Later, attention-based communication frameworks introduce content-based addressing to decide “who to listen to” and “what to aggregate,” improving scalability and enabling selective information sharing in larger teams [9,10]. Graph neural networks and message-passing architectures similarly model inter-agent interactions as relational aggregation over dynamically defined neighborhoods, which is well-suited for variable-sized agent populations and structured cooperation [1,2,9,10]. While these methods demonstrate that explicit information exchange can substantially improve coordination, many of them assume frequent communication and do not explicitly optimize the trade-off between message cost and cooperative performance, which can be critical in bandwidth- or latency-constrained settings [11,16,22]. This limitation motivates the study of communication-efficient and sparse communication mechanisms, which aim to reduce communication overhead while preserving coordination quality.

### 2.3. Communication Efficiency and Sparse/Event-Triggered Communication

In practical multi-agent systems, communication is often constrained by bandwidth, latency, energy, or privacy, motivating research on communication-efficient MARL  [4,9,11,16]. A representative direction is to learn when to communicate via scheduling or gating, where each agent decides whether to broadcast (or which subset of agents should communicate) under a limited budget [9,11,16]. Other approaches introduce explicit regularizers or bottlenecks on messages to reduce communication frequency, message dimensionality, or overall information flow [9,10,11]. Sparse or event-triggered communication is particularly appealing because it encourages agents to communicate only at critical decision points, which often correspond to tactical events such as target switching, engagement initiation, or coordinated retreat [9,11,16]. However, learning sparse communication is non-trivial: Naive gating can collapse to always-on or always-off behaviors, discrete triggers can lead to high-variance gradients, and excessive sparsification may remove the very signals needed for intent alignment, degrading group strategies even if individual behaviors remain locally reasonable [25,26,27]. Therefore, achieving an effective balance between communication sparsity and coordination quality remains an ongoing challenge, especially for value-based CTDE methods in partially observable cooperative tasks where emergent group strategies critically depend on timely and selective information exchange [3,5,9,10].

### 2.4. Gaps in Existing Research and the Positioning of This Work

The studies reviewed above establish three important foundations. Value-based CTDE methods provide stable joint value learning and effective credit assignment in cooperative MARL [28,29,30]. Differentiable and attention-based communication methods show that message exchange and attention-guided state abstraction can improve coordination under partial observability [31,32]. Sparse, targeted, hypergraph-based, and event-triggered methods further demonstrate that communication cost can be reduced by improving message specificity, suppressing irrelevant information, or triggering information exchange only when it is useful [33,34,35]. However, these lines of work still leave an important gap: The communication decision is often separated from the formation of the cooperative policy. In many existing approaches, communication is optimized mainly as a message-passing or scheduling layer. The downstream policy may receive messages, but the method does not explicitly show how communication changes individual utility estimation, joint value aggregation, or the resulting coordination pattern. Dense communication is expressive but can introduce redundant and task-irrelevant context. Selective or sparse communication reduces the number of messages, but its main objective is often communication efficiency rather than policy shaping. Event-triggered communication can activate messages around state changes, but the trigger is not always coupled with a communication-aware value-decomposition pathway or behavior-level analysis.

Consequently, current sparse and selective communication models do not fully explain how the retained messages are incorporated into a shared cooperative policy under CTDE [30,33,34]. They may reduce the frequency of communication, but message sparsity alone does not guarantee that the remaining information contributes to target selection, formation control, attack timing, or other coordinated behaviors. This limitation is particularly important for SMAC-style micromanagement tasks, where coordination quality depends not only on whether agents exchange information, but also on whether the exchanged information affects the individual utilities that drive decentralized action selection.

This work addresses the above gap by positioning sparse communication as a policy-shaping component within a value-decomposition framework. Specifically, the proposed method first uses a learned threshold-gated trigger to suppress redundant transmissions, then applies a neighborhood-constrained attention mechanism to aggregate only activated and relevant neighbor messages. The resulting communication context is fused into the recurrent utility-estimation pathway, and the QMIX mixer aggregates these communication-aware individual utilities into the team value. Compared with dense communication, the proposed framework avoids indiscriminate message broadcasting. Compared with sparse, selective, or event-triggered schemes that mainly emphasize communication reduction [33,34,35], our method explicitly connects communication to utility estimation and further evaluates its effect on behavior-level coordination patterns.

## 3. Method

This section presents the proposed communication framework, including the overall communication architecture (Section 3.1), the visual-encoding-based trigger module (Section 3.2), and the communication-aware QMIX formulation (Section 3.3). The detailed network configurations are summarized in Appendix A Table A1.

### 3.1. Communication Setting and Overall Framework

**Figure 1:**
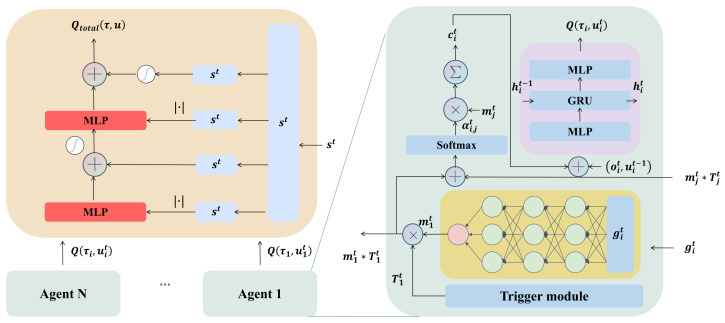
Overall architecture of the proposed communication-aware QMIX framework. The pipeline consists of local observation encoding, communication mask construction, trigger-based message gating, neighbor-constrained attention aggregation, recurrent individual utility estimation, and QMIX value mixing. Figure 2 further details the internal process from fused trigger representation to binary communication activation.

In cooperative multi-agent settings, agents operate under partial observability, where each agent only has access to local observations. Communication enables agents to exchange complementary information and improve coordination. However, unrestricted communication often introduces redundant interactions and unnecessary communication overhead. To address this issue, we design a neighbor-sparse communication mechanism with trigger-based message transmission and threshold-based regularization, so that nearby agents communicate selectively while excessive communication is discouraged during training. As illustrated in Figure 1, the proposed framework integrates sparse communication, message aggregation, recurrent utility estimation, and value decomposition into a unified architecture.

Before introducing the neural communication architecture, we first define the underlying communication setting. Consider a cooperative system with *N* agents. At time step *t*, the agents form a directed communication graph $G^{t}=\left(V,E^{t}\right)$, where each node corresponds to an agent and each directed edge indicates that one agent is allowed to transmit information to another. A dense communication baseline can be represented by the full adjacency mask

(1) $$A_{ij}^{\mathrm{full}}=I\left(i\neq j\right),$$

where every agent can send a message to every other agent at each decision step. Under this setting, all potential communication links are active, which maximizes information exchange but may also introduce redundant and task-irrelevant messages.

In contrast, the proposed framework decomposes communication availability into two factors: a physical neighborhood constraint and a learned trigger decision. The neighborhood constraint determines whether two agents are spatially close enough to communicate, while the trigger decision determines whether the sender actually transmits a message. Therefore, the effective communication link from agent *j* to agent *i* is active only when both conditions are satisfied. This design can be viewed as applying a structured sparse mask on top of the dense communication graph, rather than assuming unrestricted message broadcasting.

**Figure 2:**
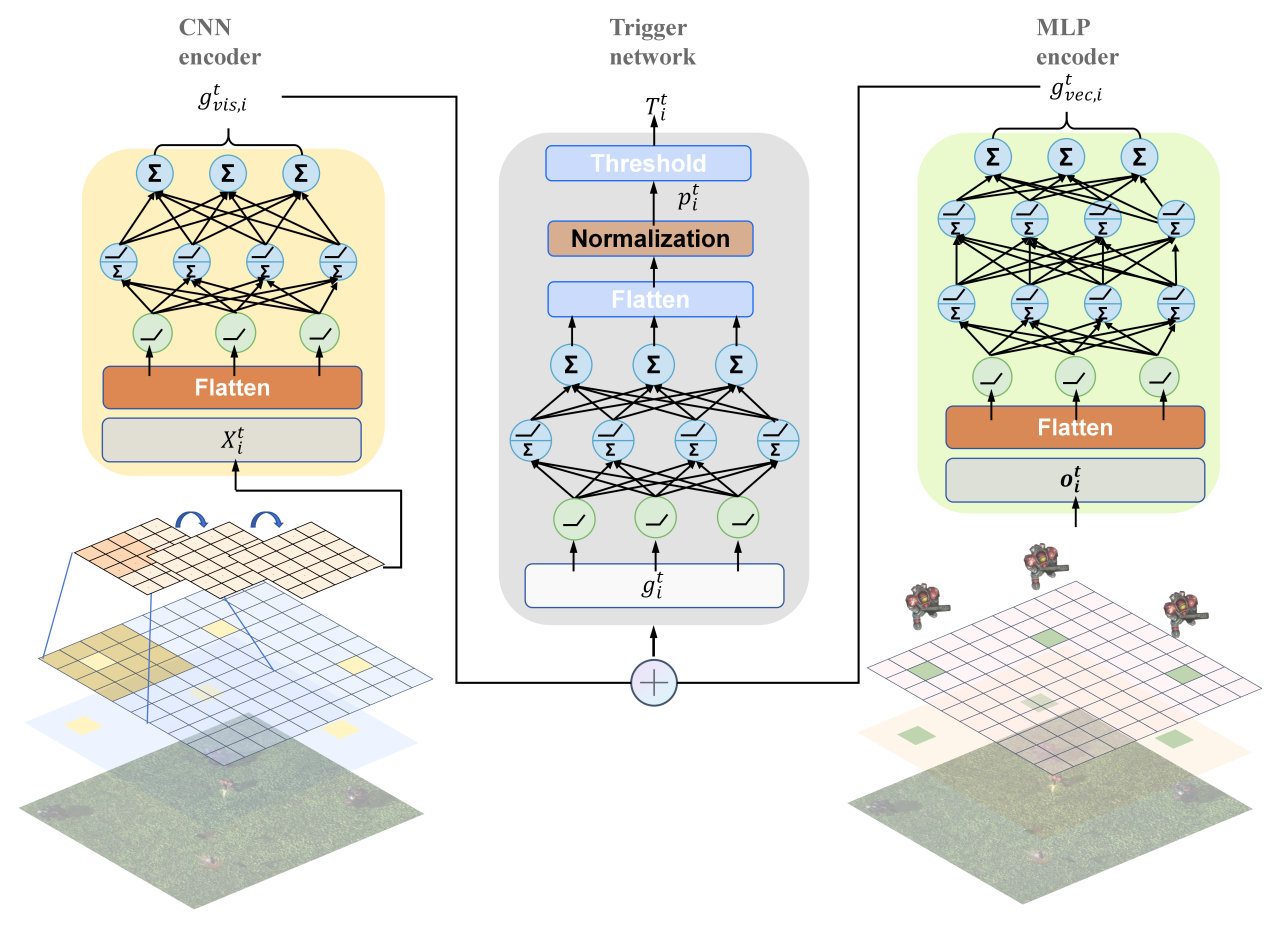
Trigger module architecture. Spatial occupancy and local vector observations are encoded and fused into a trigger representation. The fused representation is mapped to a continuous activation score, which is converted into a binary communication trigger through a hard threshold function. During training, the non-differentiable threshold operation is optimized using a straight-through estimator.

Formally, the proposed effective communication mask is factorized as

(2) $${\hat{M}}_{ij}^{t}=A_{ij}^{\mathrm{full}}M_{ij}^{t}T_{j}^{t},$$

where $M_{ij}^{t}$ denotes the spatial neighborhood mask and $T_{j}^{t}$ is the binary trigger of sender agent *j*. The dense baseline corresponds to the special case where $M_{ij}^{t}=1$ and $T_{j}^{t}=1$ for all i≠j. By contrast, our method suppresses communication either when the receiver and sender are outside the local communication range or when the sender’s trigger is inactive. This formulation makes explicit the difference between dense communication and the proposed threshold-gated sparse communication mechanism.

At each time step *t*, each agent *i* maintains an action–observation history $\tau_{i}^{t}=\left(o_{i}^{1:t},u_{i}^{0:t-1}\right),$ where $o_{i}^{t}$ denotes the local observation vector and $u_{i}^{t}$ denotes the executed action. For the first decision step, the previous action input is initialized as a zero vector. Based on its local history, each agent estimates an individual action value function for decentralized decision-making.

Unless otherwise stated, all agents share the parameters of the local encoder, message encoder, trigger-based communication module, recurrent utility network, and action value head. This parameter-sharing scheme improves sample efficiency and is standard in cooperative MARL.

#### 3.1.1. Neighbor-Based Sparse Communication

To reduce communication overhead and avoid redundant interactions, communication is restricted to a dynamic local neighbor set determined by spatial proximity. Specifically, at each time step *t*, agents within a predefined communication radius of agent *i* are considered its neighbors. We define a binary adjacency mask

(3) $$M_{ij}^{t}=I\;\left(d_{ij}^{t}\leq r,\;i\neq j\right),$$

where $d_{ij}^{t}$ is the spatial distance between agents *i* and *j* at time *t*, *r* is the communication radius, and $M_{ij}^{t}=1$ indicates that agent *i* can receive information from agent *j* at time *t*. Here, I(·) denotes the indicator function, which equals 1 if the condition is satisfied and 0 otherwise.

Each agent first encodes its current local observation and previous action into a feature representation as

(4) $$z_{i}^{t}=f_{\mathrm{emb}}\;\left([o_{i}^{t},u_{i}^{t-1}]\right),$$

where $f_{\mathrm{emb}}\left(\cdot \right)$ denotes the local feature encoder and $z_{i}^{t}\in R^{d_{z}}$ is the resulting feature vector.

Based on this representation, a message embedding is generated as

(5) $$m_{i}^{t}=f_{\mathrm{msg}}\left(z_{i}^{t};\theta_{\mathrm{msg}}\right),$$

where $f_{\mathrm{msg}}\left(\cdot \right)$ is the message encoder and $m_{i}^{t}\in R^{d_{m}}$ denotes the outgoing message vector.

To enable adaptive communication, we introduce a binary trigger variable $T_{i}^{t}\in \left\{0,1\right\}$, which is described in detail in Section 3.2. It determines whether agent *i* transmits its message. The gated transmitted message is defined as

(6) $${\tilde{m}}_{i}^{t}=T_{i}^{t}\cdot m_{i}^{t}.$$

Combining neighborhood sparsity and trigger gating yields the effective communication mask

(7) $${\hat{M}}_{ij}^{t}=M_{ij}^{t}\cdot T_{j}^{t},$$

where ${\hat{M}}_{ij}^{t}=1$ indicates that agent *i* receives the message from agent *j* at time *t*. Accordingly, the effective sender set for receiver *i* is

(8) $$N_{i}^{t}=\left\{j|{\hat{M}}_{ij}^{t}=1\right\}.$$

#### 3.1.2. Message Aggregation and Fusion

For each receiver agent *i*, incoming messages from effective neighbors are aggregated into a communication context vector $c_{i}^{t}$. Since different neighbors may provide information of different relevance, we adopt an attention-based aggregation mechanism as

(9) $$\begin{array}{cc} e_{ij}^{t} & =\psi \;\left([z_{i}^{t},{\tilde{m}}_{j}^{t}]\right), \end{array}$$

(10) $$\begin{array}{cc} \alpha_{ij}^{t} & =\frac{\exp\left(e_{ij}^{t}\right){\hat{M}}_{ij}^{t}}{\sum_{k\neq i}\exp\left(e_{ik}^{t}\right){\hat{M}}_{ik}^{t}}, \end{array}$$

(11) $$\begin{array}{cc} c_{i}^{t} & =\sum_{j\neq i}\alpha_{ij}^{t}{\tilde{m}}_{j}^{t}, \end{array}$$

where ψ(·) is a scoring network that maps the concatenated feature $\left[z_{i}^{t},{\tilde{m}}_{j}^{t}\right]$ to a scalar attention logit, and $\alpha_{ij}^{t}$ denotes the normalized attention weight. The scalar $e_{ij}^{t}$ represents the unnormalized attention score measuring the relevance of the message from agent *j* to agent *i* at time *t*. The aggregation is performed only over the valid incoming senders indicated by ${\hat{M}}_{ij}^{t}$. When at least one valid incoming sender exists, the attention weights are normalized as above; otherwise, we directly set $c_{i}^{t}=0,$ which represents the null communication context.

The agent recurrent state is then updated by

(12) $$h_{i}^{t}=\mathrm{GRU}\;\left(h_{i}^{t-1},\left[z_{i}^{t},c_{i}^{t}\right]\right),$$

where $h_{i}^{t}\in R^{d_{h}}$ denotes the hidden state vector. The individual utility network outputs the Q-values over the discrete action space as

(13) $$q_{i}^{t}=f_{Q}\left(h_{i}^{t};\theta_{Q}\right),$$

and the utility corresponding to the executed action $u_{i}^{t}$ is

(14) $$Q_{i}\left(\tau_{i}^{t},u_{i}^{t}\right)=q_{i}^{t}\left[u_{i}^{t}\right].$$

During decentralized execution, each agent computes its local feature representation, trigger activation, communicated message, aggregated communication context, and action values using only its local observation history and the messages received from neighboring agents.

### 3.2. Trigger-Based Communication Module

To further reduce redundant communication, we introduce a trigger-based communication module together with an excess-activation regularization mechanism, so that message transmission is learned adaptively while excessive communication is explicitly discouraged during training.

Each agent constructs a multi-modal trigger representation based on (i) a spatial occupancy map of nearby allies and (ii) its local vector observation. Specifically, the occupancy of neighboring allied agents is encoded as a binary map $X_{i}^{t}\in {\left\{0,1\right\}}^{31\times 31}$, where the map is centered at agent *i*, and each grid cell indicates whether a neighboring ally occupies the corresponding relative spatial location. The map size 31×31 is chosen to provide a fixed-resolution coverage of the agent-centered local perceptual region in SMAC while keeping the visual encoder lightweight. If the local perceptual region exceeds the map boundary, the valid observable area is clipped by the fixed 31×31 boundary.

A CNN encoder extracts spatial features as

(15) $$g_{\mathrm{vis},i}^{t}=f_{\mathrm{cnn}}\left(X_{i}^{t};\theta_{\mathrm{cnn}}\right),$$

where $g_{\mathrm{vis},i}^{t}\in R^{d_{\mathrm{vis}}}$ is the visual feature vector. In parallel, the local vector observation is encoded as

(16) $$g_{\mathrm{vec},i}^{t}=f_{\mathrm{mlp}}\left(o_{i}^{t};\theta_{\mathrm{vec}}\right),$$

where $g_{\mathrm{vec},i}^{t}\in R^{d_{\mathrm{vec}}}$ is the vector feature representation. The two representations are fused into a unified trigger feature as

(17) $$g_{i}^{t}=\phi \;\left([g_{\mathrm{vec},i}^{t},g_{\mathrm{vis},i}^{t}]\right),$$

where ϕ(·) is a linear projection layer.

The trigger head first maps the fused representation to a scalar logit:

(18) $$a_{i}^{t}=f_{\mathrm{tri}}\left(g_{i}^{t}\right),$$

where $a_{i}^{t}$ denotes the unnormalized trigger logit. The logit is then converted into a continuous activation score through a sigmoid function:

(19) $$p_{i}^{t}=\sigma \left(a_{i}^{t}\right),$$

where $p_{i}^{t}\in \left(0,1\right)$ represents the communication activation score. A binary communication trigger is obtained by applying a hard threshold function:

(20) $$T_{i}^{t}=H\left(p_{i}^{t}-0.5\right),$$

where H(·) is the Heaviside step function, defined as

(21) $$H\left(x\right)=\left\{\begin{array}{cc} 1, & x>0,\\ 0, & x\leq 0. \end{array}\right.$$

Thus, $T_{i}^{t}=1$ means that agent *i* transmits its message at time step *t*, while $T_{i}^{t}=0$ means that the message is suppressed. We adopt 0.5 as the decision boundary because $p_{i}^{t}$ is produced by a sigmoid function and therefore has a natural midpoint between inactive and active communication states.

The hard threshold function is non-differentiable and has zero gradients almost everywhere. To enable end-to-end training, we adopt a straight-through estimator (STE). In the forward pass, the model uses the hard binary trigger $T_{i}^{t}=H\left(p_{i}^{t}-0.5\right)$. In the backward pass, the gradient of the hard threshold is approximated by the identity mapping:

(22) $$\frac{\partial T_{i}^{t}}{\partial p_{i}^{t}}\approx 1.$$

Equivalently, this can be interpreted as using the discrete trigger for message transmission while allowing gradients to flow through the continuous activation score during optimization. As a result, the trigger module can receive gradients from the temporal-difference loss through the communication-dependent utility estimation pathway:

(23) $$\frac{\partial L_{\mathrm{TD}}}{\partial \theta_{\mathrm{tri}}}\approx \frac{\partial L_{\mathrm{TD}}}{\partial T_{i}^{t}}\frac{\partial p_{i}^{t}}{\partial \theta_{\mathrm{tri}}}.$$

It is important to distinguish the binary decision boundary from the regularization threshold. The value 0.5 is used only to convert the sigmoid activation score into a binary trigger. By contrast, the parameter δ is not used for hard trigger generation. Instead, δ appears in the communication regularization term and determines the activation level above which excessive communication is penalized. Therefore, the proposed design decouples message transmission from communication regularization: $T_{i}^{t}$ controls whether communication occurs in the forward pass, whereas δ controls how strongly high activation scores are discouraged during training.

The trigger network is optimized through two coupled pathways. First, the temporal-difference loss backpropagates through the communication-dependent utility estimation path, since the binary trigger affects message transmission, message aggregation, and ultimately the individual utilities and joint action value. Second, the excess-activation regularizer directly penalizes large activation scores $p_{i}^{t}$. The STE enables gradients from the TD loss to pass through the non-differentiable binary trigger, while the regularization term provides an additional continuous supervision signal for controlling communication tendency.

### 3.3. Communication-Augmented QMIX

We adopt a QMIX-based value decomposition framework under centralized training and decentralized execution (CTDE) [5]. The joint action value function is defined as

(24) $$Q_{\mathrm{tot}}\left(\tau^{t},u^{t}\right)=f_{\mathrm{mix}}\;\left({\left\{Q_{i}\left(\tau_{i}^{t},u_{i}^{t}\right)\right\}}_{i=1}^{N},s^{t};\theta_{\mathrm{mix}}\right),$$

where $s^{t}$ denotes the global state vector available only during training, and $f_{\mathrm{mix}}\left(\cdot \right)$ is the mixing network constrained to satisfy the monotonicity condition

(25) $$\frac{\partial Q_{\mathrm{tot}}}{\partial Q_{i}}\geq 0,\;\forall i.$$

Due to the monotonic mixing constraint, the maximizing joint action can be obtained by independently selecting the greedy action of each agent. Let

(26) $$u_{i}^{⋆}=\arg\max_{u_{i}}Q_{i}\left(\tau_{i}^{t+1},u_{i}\right),$$

and denote the corresponding joint greedy action as $u^{⋆}=\left(u_{1}^{⋆},\ldots ,u_{N}^{⋆}\right)$. Then the TD target is written as

(27) $$y^{t}=r^{t}+\gamma \left(1-d^{t}\right)\;Q_{\mathrm{tot}}^{-}\left(\tau^{t+1},u^{⋆}\right),$$

where $r^{t}$ is the team reward, γ is the discount factor, $d^{t}\in \left\{0,1\right\}$ is the episode termination indicator, and $Q_{\mathrm{tot}}^{-}$ denotes the target network.

The temporal-difference loss is

(28) $$L_{\mathrm{TD}}=E_{B}\left[{\left(y^{t}-Q_{\mathrm{tot}}\left(\tau^{t},u^{t}\right)\right)}^{2}\right],$$

where B denotes the replay batch.

To encourage communication efficiency without over-suppressing useful message exchange, we introduce a communication regularization term based on excess trigger activation as

(29) $$L_{\mathrm{comm}}=E_{B}\left[\frac{1}{NH}\sum_{t=1}^{H}\sum_{i=1}^{N}\max\left(0,\;p_{i}^{t}-\delta \right)\right],$$

where *H* is the episode horizon, *N* is the number of agents, and δ∈(0,1) is a prescribed activation threshold. Under this design, trigger activations below δ incur no additional penalty, while activations above δ are penalized proportionally to their excess magnitude. The threshold δ is a scenario-dependent hyperparameter controlling the activation level at which communication is penalized. Its concrete values and selection strategy are described in detail in Section 4. Since the regularization is applied to the continuous activation score $p_{i}^{t}$ rather than the binary trigger $T_{i}^{t}$, it provides a smoother optimization signal for controlling communication intensity. Therefore, a higher δ corresponds to a looser communication constraint, since a larger activation range is exempt from regularization.

The empirical communication rate is computed as the average proportion of active triggers over all agents and time steps:

(30) $$\rho_{\mathrm{comm}}=\frac{1}{NH}\sum_{t=1}^{H}\sum_{i=1}^{N}T_{i}^{t}.$$

This metric measures the realized frequency of message transmission during execution, while $L_{\mathrm{comm}}$ regularizes the continuous activation scores during training. This evaluation indicator was used to assess communication efficiency in Section 4.1 and  Section 4.3.

The overall training objective is

(31) $$L=L_{\mathrm{TD}}+\lambda_{\mathrm{comm}}L_{\mathrm{comm}},$$

where $\lambda_{\mathrm{comm}}$ controls the trade-off between task performance and communication efficiency.

## 4. Experiments

We evaluate the proposed method on the StarCraft II Multi-Agent Challenge (SMAC) benchmark, based on StarCraft II version 4.10, a cooperative micromanagement benchmark where each learning agent controls one allied unit and coordinates with teammates to defeat enemy units under partial observability. The experiments are conducted on four representative scenarios: 2s3z, where two allied Stalkers and three allied Zealots fight against an enemy team with the same unit composition; 10m_vs_11m, where ten allied Marines fight against eleven enemy Marines; MMM, where a heterogeneous team composed of Marines, Marauders, and a Medivac fights against a comparable enemy group; and 1c3s5z, where one Colossus, three Stalkers, and five Zealots form a heterogeneous combat group.

These scenarios cover homogeneous and heterogeneous unit compositions, symmetric and asymmetric combat settings, and different coordination difficulties. The 2s3z scenario is a symmetric mixed-unit task, where agents must coordinate ranged Stalkers and melee Zealots, making it suitable for evaluating formation control, target selection, and cooperation between units with different roles. The 10m_vs_11m scenario tests a homogeneous Marine team under numerical disadvantage, where successful policies require concentrated fire, spacing control, and effective attack–move transitions. The MMM and 1c3s5z scenarios further emphasize heterogeneous role coordination: MMM requires cooperation between damage-dealing Marines and Marauders and the healing Medivac, while 1c3s5z evaluates front-line protection, long-range damage support, and coordinated engagement among units with different attack ranges and tactical functions.

All learning curves are evaluated every 10k environment steps using test episodes, and the shaded areas in the figures denote the standard deviation across runs. Depending on the scenario, the total training budget ranges from 1.2 M to 2.05 M environment steps. For scalar summaries, we report the average over the last 80 evaluations. To improve reproducibility, the main simulation settings and hyperparameters, including random seeds, software version, communication radius, message dimension, learning rate, batch size, replay buffer size, and the communication coefficient $\lambda_{\mathrm{comm}}$, are summarized in Appendix A Table A1.

To study both task-level performance and behavior-level policy variation under communication control, we carry out three groups of experiments: multi-map performance comparison, communication effect evaluation, and ablation analysis. The results are reported using both task-level metrics and behavior-level metrics.

For task-level evaluation, we use win rate as the primary performance metric. For behavior-level evaluation, we consider several interpretable metrics that characterize coordination and micro-control behaviors. Unless otherwise stated, all reported behavior metrics are computed from test episodes at every 10k training steps and summarized using the last 80 evaluation records.

Minimum separation mean measures local spatial coordination:

(32) $${\mathrm{minsep}}_{\mathrm{mean}}=\frac{1}{T}\sum_{t=1}^{T}\frac{1}{A}\sum_{i=1}^{A}\min_{j\neq i}{∥x_{i}^{t}-x_{j}^{t}∥}_{2}.$$

Here, *T* denotes the total time steps, *A* is the number of agents, and $x_{i}^{t}$ is the position vector of agent *i* at time *t*. Larger values indicate more dispersed formations.

Focus-fire quantifies coordinated targeting:

(33) $${\mathrm{FocusFire}}_{\mathrm{mean}}=\frac{1}{|T_{\mathrm{attack}}|}\sum_{t\in T_{\mathrm{attack}}}\max_{j}\sum_{i=1}^{A}I\left(a_{i}^{t}=j\right).$$

Here, $T_{\mathrm{attack}}$ is the set of attack time steps, and $a_{i}^{t}$ denotes the action executed by agent *i* at time *t*. When $a_{i}^{t}=j$, agent *i* attacks enemy *j* at time *t*. Higher values indicate stronger coordination.

Target entropy evaluates target diversity:

(34) $${\mathrm{Entropy}}_{\mathrm{mean}}=\frac{1}{|T_{\mathrm{attack}}|}\sum_{t\in T_{\mathrm{attack}}}-\sum_{j=1}^{E}\rho_{j}^{t}\log\rho_{j}^{t}/\log\left(E\right).$$

Here, *E* is the number of enemies, and $\rho_{j}^{t}$ is the proportion of agents attacking enemy *j* at time *t*. Lower values indicate more concentrated targeting. In our logs, this metric is reported as bucket_entropy.

Attack_move ratio measures micro-control behavior:

(35) $$\mathrm{AttackMoveRatio}=\frac{\sum_{t=2}^{T}\sum_{i=1}^{A}I\left({\mathrm{Attack}}_{i}^{t-1}\wedge {\mathrm{Move}}_{i}^{t}\right)}{\sum_{t=2}^{T}\sum_{i=1}^{A}I\left({\mathrm{Attack}}_{i}^{t-1}\right)+\epsilon }.$$

Here, ϵ is a small constant to avoid division by zero, and ${\mathrm{Attack}}_{i}^{t}$ and ${\mathrm{Move}}_{i}^{t}$ denote indicator variables of whether agent *i* executes an attack action or a movement action at time *t*, respectively. Higher values indicate more effective attack_move ratio transitions.

To study communication under controlled regimes, we introduce a threshold-based excess-activation penalty calibrated from the steady-state statistics of trigger activations. Specifically, for each scenario, we first run a reference setting to estimate the stable-phase trigger activation level after convergence, and denote the corresponding mean activation rate by $\mu_{s}$. We use the steady-state activation statistics only as a calibration reference for selecting scenario-specific thresholds, rather than as part of training. Based on this calibration anchor, we define three threshold settings:

(36) $$\delta_{\mathrm{low}}=\mu_{s},\delta_{\mathrm{mid}}=\mu_{s}+0.05,\delta_{\mathrm{high}}=\mu_{s}+0.1.$$

Under the proposed excess-activation penalty design, communication is penalized only when the trigger activation exceeds the prescribed threshold. Therefore, a higher threshold corresponds to a looser communication constraint and typically leads to a higher realized communication rate.

Table 1 summarizes the communication threshold statistics used in the experiments. Across the four SMAC scenarios, the estimated stable-phase mean activation rate $\mu_{s}$ remains relatively concentrated, ranging from 0.298 on 2s3z to 0.308 on MMM. Based on these scenario-specific calibration anchors, we construct three comparable communication regimes, namely $\delta_{\mathrm{low}}$, $\delta_{\mathrm{mid}}$, and $\delta_{\mathrm{high}}$, which are used throughout the subsequent experiments.

To ensure meaningful separation between communication regimes, we further carry out a preliminary sensitivity analysis around $\mu_{s}$. In this analysis, the threshold offset is applied relative to $\mu_{s}$, and ally minimum separation (ally_minsep) is used as the primary diagnostic metric because it most directly reflects formation-level coordination and spatial control.

As shown in Table 2, offsets around 0.05 and 0.10 yield distinguishable yet stable changes in formation behavior, while larger offsets do not consistently provide further separation. Therefore, we adopt 0.05 and 0.10 as the two positive threshold offsets. We do not claim that these offsets are universally optimal; rather, they are used to define reproducible communication regimes for comparative analysis.

We then assess our method under these three regimes and compare it with the original QMIX baseline without explicit communication control, enabling a systematic analysis of the trade-off between communication cost and task performance.

### 4.1. Performance on Multi-Map Tasks

**Figure 3:**
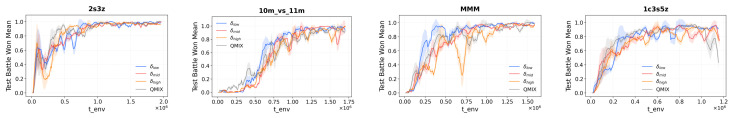
Training win rate under different communication thresholds on four SMAC scenarios: 2s3z, 10m_vs_11m, MMM, and 1c3s5z. Curves are evaluated every 10k environment steps using Test episodes, and shaded areas denote the standard deviation across runs.

To study whether communication control can preserve performance while reducing communication, we first carry out a multi-map comparison across the four SMAC scenarios using win rate as the primary evaluation metric. Figure 3 illustrates the training dynamics under different communication thresholds, while Table 3 reports the final win rate averaged over the last 80 evaluation records.

The results show that all threshold settings converge to competitive performance across the four scenarios. While the learning curves differ in stability, the final win rates remain comparable, indicating that communication control does not degrade overall task performance. As shown in Table 3, several threshold-controlled settings outperform the QMIX baseline.

Specifically, on 2s3z, the proposed method achieves a best win rate of 0.974 under $\delta_{\mathrm{high}}$, slightly improving over the QMIX baseline (0.972) and outperforming the mid-threshold setting (0.966). On MMM, the improvement is more pronounced: The low-threshold setting reaches 0.966, higher than the QMIX baseline (0.921), corresponding to a relative gain of approximately 4.5%. On 10m_vs_11m, the best performance is achieved at $\delta_{\mathrm{mid}}$ with a win rate of 0.884, compared to 0.856 for QMIX. Similarly, on 1c3s5z, the highest win rate (0.955) is obtained under $\delta_{\mathrm{high}}$, exceeding the baseline performance (0.941). Overall, these results demonstrate that the proposed communication mechanism not only maintains competitive performance across different thresholds but can also provide consistent improvements over the baseline in several scenarios, especially in more challenging tasks such as MMM and 10m_vs_11m.

In addition, high-threshold settings (corresponding to higher communication frequency under the proposed excess-activation penalty design) tend to exhibit more pronounced fluctuations during training. This effect is especially visible in maps such as MMM and 2s3z, where the corresponding curves show noticeable oscillations before convergence. We attribute this phenomenon to the presence of redundant or noisy information under high communication intensity, which can interfere with stable policy updates. In contrast, lower-threshold settings impose stronger communication suppression, often leading to smoother training dynamics and more stable convergence behavior. These observations further support that effective coordination benefits from controlled, rather than excessive, communication.

**Figure 4:**
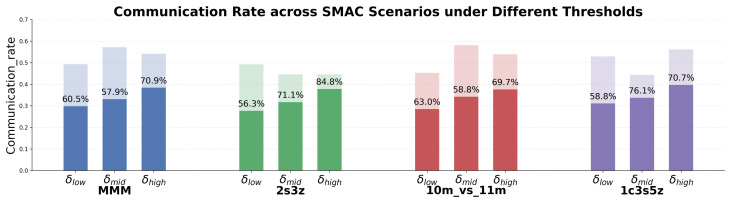
Average communication rate under different threshold settings across four SMAC scenarios.

To further study communication efficiency under the same threshold settings, we report the realized communication rate in Figure 4. The results show that the proposed threshold-based excess-activation penalty produces clearly distinguishable communication patterns across all scenarios.

Specifically, on MMM, the communication rate increases from 0.298 to 0.384 across different threshold settings; on 2s3z, it ranges from 0.277 to 0.378; on 10m_vs_11m, from 0.285 to 0.376; and on 1c3s5z, from 0.311 to 0.397. Since a higher threshold delays the onset of regularization, these results are consistent with the intended behavior of the proposed penalty design. Despite variations across tasks, the overall communication level remains consistently moderate (approximately 0.27–0.40), indicating that the proposed mechanism effectively avoids excessive message passing.

Importantly, when compared to the reference activation levels (shown by the background bars), the effective communication rate is reduced by a substantial margin. Here, the retained communication ratio is defined as the realized communication rate normalized by the corresponding reference activation level. The retained communication ratio is typically around 56% to 76%, demonstrating that a significant portion of potential communication is filtered out by the trigger mechanism.

Combining these observations with the win rate results, we find that reducing communication does not degrade performance. On the contrary, several settings with lower communication frequency achieve equal or better performance than the baseline. This suggests that the proposed threshold mechanism successfully filters out redundant interactions while preserving critical information exchange, leading to more efficient and stable multi-agent coordination.

### 4.2. Communication Effect Evaluation

**Figure 5:**
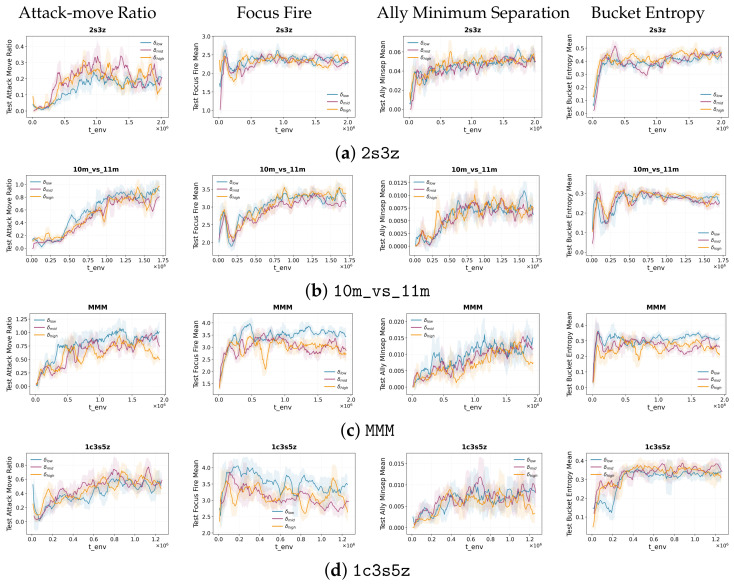
Strategy metrics under different communication thresholds. The whole figure is organized into four scenario-level subfigures: (**a**) 2s3z, (**b**) 10m_vs_11m, (**c**) MMM, and (**d**) 1c3s5z. Within each scenario-level subfigure, the four second-level panels report attack-move ratio, focus fire, ally minimum separation, and bucket entropy from left to right. Curves are evaluated every 10k environment steps on test episodes, and shaded areas denote the standard deviation.

To study how communication control changes the learned policy beyond task-level win rate, we carry out a behavior-level analysis using focus-fire, bucket entropy, ally minimum separation, and attack_move ratio as complementary evaluation metrics. Figure 5 reports the corresponding results across the four SMAC scenarios.

#### 4.2.1. Threshold-Dependent Policy Shaping Analysis

The results show that communication control leads to distinguishable policy variations across multiple behavioral dimensions. To provide a more compact quantitative summary, Table 4 reports the converged behavior-level statistics under different communication thresholds. The values are estimated from the stable segments of Figure 5 and summarized as mean ± standard deviation.

Table 4 provides a compact statistical summary of the behavior-level coordination metrics under different communication thresholds. These metrics are used to interpret how threshold-controlled sparse communication affects the learned coordination policy rather than merely reducing the number of transmitted messages. Specifically, the attack_move ratio reflects the agents’ tendency to engage in combat actions, focus-fire measures whether multiple agents concentrate attacks on common targets, ally minimum separation reflects formation spacing and local collision-avoidance behavior, and bucket entropy measures the dispersion of target selection. Therefore, changes in these metrics provide behavioral evidence of how different communication thresholds reshape cooperative policies.

It should be noted that the threshold parameter δ controls the strength of excess-activation regularization rather than the hard binary decision boundary of the trigger. A smaller δ imposes a stronger penalty on high communication activation scores, thereby encouraging more selective and sparse communication. In this regime, agents are forced to rely more heavily on locally salient and behaviorally necessary messages. A larger δ relaxes this regularization and allows more communication signals to enter the utility-estimation pathway. As a result, different threshold levels change the amount and type of information incorporated into the recurrent hidden state, which further affects individual utility estimation and the resulting decentralized action choices.

The statistical trends in Table 4 show that the effect of threshold selection is scenario-dependent. In the relatively simple 2s3z scenario, the three thresholds produce very similar attack–move and focus-fire values, with focus-fire remaining around 2.35–2.36. The small variation across thresholds indicates that this scenario has relatively low sensitivity to communication sparsity, since the required cooperative behavior can be learned with limited additional communication. This suggests that sparse communication mainly reduces redundant information exchange in simple coordination tasks without substantially changing the learned policy structure.

In contrast, more complex scenarios exhibit clearer threshold-dependent behavioral changes. In 10m_vs_11m, $\delta_{\mathrm{high}}$ achieves the highest focus-fire score (3.36±0.17), while $\delta_{\mathrm{mid}}$ obtains the lowest bucket entropy (0.26±0.02). This indicates that relaxing communication regularization can strengthen shared target information and improve concentrated attack behavior, whereas a moderate threshold can produce more stable target-selection concentration. In MMM, $\delta_{\mathrm{low}}$ yields the highest attack_move ratio (0.94±0.10) and focus-fire score (3.47±0.25), suggesting that stronger communication sparsity can encourage agents to preserve only highly task-relevant signals and execute more decisive local combat behaviors. However, $\delta_{\mathrm{high}}$ produces the lowest bucket entropy (0.24±0.03), indicating that a more relaxed communication regime can help align target selection across heterogeneous agents. In 1c3s5z, $\delta_{\mathrm{high}}$ improves several coordination indicators, including focus-fire (3.25±0.25), ally minimum separation (0.0082±0.0010), and bucket entropy (0.33±0.04), implying that this heterogeneous scenario benefits from richer communication context when coordinating different unit types.

These observations suggest a mechanism-level interpretation of policy shaping. Lower thresholds impose stronger communication regularization and encourage agents to transmit only behaviorally salient information, which can promote compact and decisive local coordination. Middle thresholds provide a compromise between communication suppression and information sharing, often yielding stable target-selection behavior. Higher thresholds allow more communication information to be incorporated into the utility-estimation process, which can improve global target alignment and heterogeneous unit coordination in more difficult scenarios. Therefore, sparse communication affects the policy not only by changing communication frequency, but also by changing which information is available when agents estimate their utilities and select decentralized actions.

Overall, the results support the claim that threshold-controlled sparse communication reshapes coordination behavior in a scenario-dependent manner. The behavioral differences across attack tendency, focus-fire, formation spacing, and target-selection entropy provide empirical evidence that the communication threshold influences the learned policy structure. However, the effect is not strictly monotonic across all metrics or scenarios. Instead, different thresholds emphasize different dimensions of coordination, indicating that the threshold should be regarded as a behavior-shaping factor that balances communication efficiency, local decision decisiveness, and global cooperative alignment.

#### 4.2.2. Effect of Task Complexity Across Maps

To study whether the benefit of communication control depends on task difficulty, we compare the performance and behavioral spread across maps of different complexity. The results show that the impact of communication becomes more pronounced as task complexity increases.

In 2s3z, the performance gap between the best and worst threshold settings is small (0.974 vs. 0.966, gap 0.008), whereas in 10m_vs_11m, the gap is substantially larger (0.884 vs. 0.856, gap 0.028). Behavioral differences follow the same trend. In 2s3z, focus-fire varies within a relatively narrow range of approximately 2.420–2.507, while in 10m_vs_11m it spans roughly 3.126–3.414. Similarly, the attack–move ratio exhibits a visibly larger spread in the more complex scenarios.

These results indicate that communication control is increasingly critical in complex tasks, where improper communication can more easily degrade coordination quality. In contrast, selective communication enables more stable and effective policies as coordination demands grow.

### 4.3. Ablation Study

#### 4.3.1. Effect of Disabling Communication

To study the contribution of the communication mechanism itself, we introduce a static no-communication control, where the communication module remains in the architecture but its output is fixed to zero by forcing the trigger variable to be inactive for all agents at all time steps. This keeps the network structure unchanged and ensures a fair comparison, while fully disabling observation-dependent communication.

**Figure 6:**
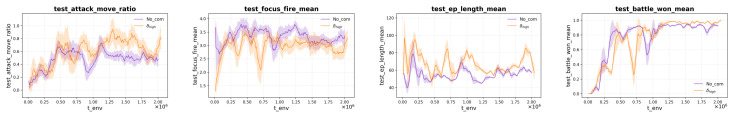
Ablation on MMM: attack–move ratio, focus-fire, episode length, and win rate under communication-enabled and no-communication settings. Curves are evaluated every 10k environment steps on test episodes, and shaded areas denote the standard deviation.

Figure 6 presents the results on the MMM scenario. At the task level, disabling communication leads to only a marginal degradation in final performance, with the win rate decreasing from approximately 0.954 to 0.935. This indicates that agents can still learn a reasonably effective policy without communication.

The results show that substantial differences emerge in behavior-level metrics even though the win-rate drop is limited. Specifically, the attack–move ratio drops markedly from approximately 0.824 to about 0.521, indicating a clear reduction in attack–move ratio micro-control. At the same time, the focus-fire value increases from around 3.355 to 3.653, suggesting that agents tend to concentrate fire more aggressively on a single target. In addition, the episode length decreases from roughly 68 to 55, reflecting shorter and less sustained engagements.

These results reveal a meaningful shift in the learned policy structure. Without communication, agents tend to adopt a simplified strategy characterized by more static positioning and direct damage exchange, leading to faster but less adaptive interactions. In contrast, communication enables agents to coordinate movement and timing, resulting in more refined micro-control and prolonged engagements.

Overall, although communication brings only limited gains in final win rate on MMM, it plays a critical role in shaping coordinated behaviors. In this ablation setting, communication mainly improves micro-control and policy quality rather than merely increasing the final task-level metric.

#### 4.3.2. Applicability Across MARL Backbones

To study whether the proposed communication framework is specific to QMIX or can be transferred to other MARL algorithms, we further integrate it into multiple backbones, including parameter-sharing Deep Q-Network (DQN) [36], Multi-Agent Proximal Policy Optimization (MAPPO) [37], QMIX [5], and Multi-Agent Deep Deterministic Policy Gradient (MADDPG) [23]. In all cases, the same communication module is inserted before action selection, providing a unified interface across value-based and actor–critic methods.

**Figure 7:**
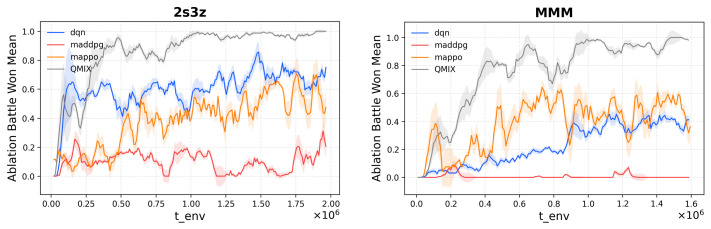
Win-rate comparison of different MARL backbones equipped with the proposed communication framework on 2s3z and MMM. Curves are evaluated every 10k environment steps on test episodes, and shaded areas denote the standard deviation.

Figure 7 shows the win-rate comparison on 2s3z and MMM. Importantly, all curves in this figure correspond to backbone methods equipped with the proposed communication framework. The results show that, across all methods, the communication-enabled variants remain trainable under identical training settings, indicating that the proposed framework is broadly compatible with different MARL backbones.

However, the effectiveness of communication-aware learning varies substantially across different backbones. In particular, QMIX consistently achieves the highest performance. On 2s3z, QMIX reaches a win rate of approximately 0.95–1.00, outperforming DQN (about 0.75–0.85), MADDPG (about 0.45–0.65), and MAPPO (about 0.10–0.30). A similar trend is observed on MMM, where QMIX maintains a win rate close to 0.90–1.00, while the other methods remain substantially lower.

These results indicate that the proposed communication framework is broadly compatible with different MARL algorithms, but its effectiveness is not uniform across learning paradigms. A plausible explanation is that QMIX, with explicit cooperative value decomposition, is better aligned with shared communication signals and therefore better able to exploit additional coordination information. By contrast, actor–critic methods such as MAPPO and MADDPG may face more difficult credit assignment and optimization instability under multi-agent interaction noise, which can limit the benefit obtained from communication. Similarly, DQN lacks an explicit coordination mechanism, which may further constrain its performance.

Overall, the results demonstrate that the proposed framework is broadly applicable, but achieves the strongest empirical gains when combined with cooperative value-based methods such as QMIX.

#### 4.3.3. Comparison with Mainstream Communication Methods

To further evaluate the effectiveness of the proposed trigger-based communication mechanism, we additionally compare our method with several representative communication-based MARL approaches, including ATOC [9], SchedNet [38], and G2ANet [39]. Following the same experimental protocol, all methods are trained under identical QMIX backbones, replay-buffer settings, training budgets, and evaluation procedures. The comparison focuses on two aspects: task performance (battle win rate) and communication efficiency (communication rate).

**Figure 8:**
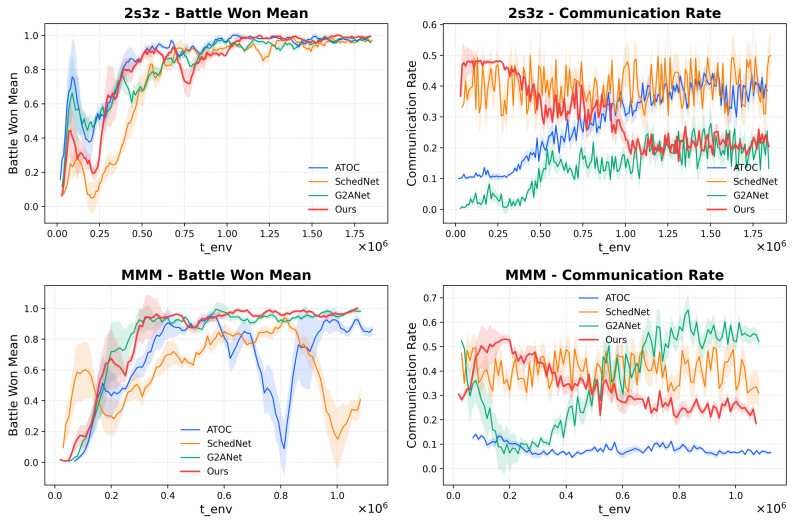
Comparison with mainstream communication-based MARL methods on the 2s3z and MMM scenarios. The first column presents the battle win rate, while the second column reports the communication rate during training.

From the results on the 2s3z map shown in Figure 8, all methods eventually converge to high win rates, but their convergence behaviors and communication costs differ significantly. ATOC achieves rapid early-stage improvement and reaches a battle win rate of approximately 0.75 at $0.1\times 10^{6}$ environment steps, outperforming SchedNet (≈0.28) and our method (≈0.45) during the initial exploration stage. However, ATOC continuously increases its communication activity throughout training, with the communication rate rising from nearly 0.10 to around 0.40 after convergence. SchedNet maintains an even higher communication rate between 0.35 and 0.50 over the entire training process. In contrast, the proposed method gradually reduces communication usage from approximately 0.48 in the early stage to around 0.20 after convergence, while still maintaining a final battle win rate close to 0.99. Compared with ATOC and SchedNet, our approach therefore achieves similar asymptotic performance using nearly half the communication frequency.

The comparison on the more challenging MMM scenario further demonstrates the robustness and communication efficiency of the proposed method. As illustrated in Figure 8, ATOC and SchedNet exhibit obvious instability during later training stages. In particular, the win rate of ATOC drops sharply to nearly 0.10 around $0.8\times 10^{6}$ environment steps, while SchedNet decreases to below 0.20 near $1.0\times 10^{6}$ steps. By contrast, our method maintains a stable win rate above 0.95 after approximately $0.35\times 10^{6}$ steps and finally converges close to 1.0. Meanwhile, the communication rate of our method decreases from approximately 0.52 to around 0.24 during training, whereas G2ANet continuously increases its communication rate to more than 0.55 in the later stage. Although G2ANet achieves competitive final performance, it requires substantially denser communication interactions. Overall, the results indicate that the proposed trigger-based communication mechanism can effectively suppress redundant communication while preserving stable cooperative behaviors and strong task performance across different SMAC scenarios.

## 5. Discussion

The results suggest that strong coordination in cooperative MARL does not strictly rely on dense or continuous communication. In practice, communication that is only triggered when necessary is often sufficient to reach comparable—if not better—performance, while noticeably reducing communication overhead. This, to some extent, implies that excessive communication may do more harm than good: Redundant or noisy signals can accumulate and disrupt stable policy learning. By contrast, selectively activated communication appears to coincide more naturally with moments that genuinely require coordination. Beyond efficiency considerations, communication also seems to play a deeper role in shaping how policies evolve. Rather than acting purely as an information channel, it influences the structure of agent behaviors. When communication is maintained at an appropriate level of sparsity, coordination becomes more organized: Focus-fire behavior is more consistent, attack–move execution appears smoother, and formation control exhibits greater stability. On the other hand, when communication occurs too frequently, training tends to become less stable, often accompanied by larger performance fluctuations. This pattern suggests that an excess of signals may interfere with the gradual formation of effective coordination strategies.

The introduction of a lightweight CNN-based spatial encoder further refines this process. By incorporating local neighborhood information—such as agent density and relative positioning—the trigger module becomes sensitive to spatial context that is directly interpretable. Consequently, communication decisions are no longer based solely on latent features but are grounded in observable structural cues. Empirically, this shift is reflected in clearer behavioral differences across communication regimes, indicating that communication can, in effect, modulate how coordination patterns take shape.

Another aspect worth noting is the explicit trade-off between communication cost and coordination quality enabled by the excess-activation penalty threshold. When the threshold is set lower, communication is more tightly constrained, often leading to a substantial reduction in communication frequency without degrading performance—and in some cases even improving it. In contrast, looser thresholds tend to allow unnecessary communication to persist, which can undermine training stability. Taken together, these observations point to a broader implication: In MARL systems, it is not merely the presence of sparse communication that matters, but how carefully that sparsity is regulated to support structured policy development.

### Limitations

Several limitations of the proposed framework should be acknowledged. To begin with, the trigger mechanism depends on manually defined hyperparameters, such as threshold values and bias offsets. These parameters may not transfer seamlessly across environments and often require additional tuning as task complexity changes. In addition, the current approach primarily captures local interactions and does not explicitly account for long-range dependencies or global coordination signals, which could become important in scenarios involving large-scale synchronization. Therefore, the conclusions of this study should be interpreted within the evaluated SMAC micromanagement scenarios. Further validation is needed before generalizing the observed benefits of threshold-gated sparse communication to cooperative MARL tasks with different communication topologies, reward structures, reward sparsity levels, or agent scales.

Finally, although the empirical results highlight a clear connection between communication and policy structure, the underlying mechanisms remain insufficiently understood. A more rigorous theoretical account of how communication influences emergent behaviors is still lacking. Future work could therefore explore directions such as adaptive threshold learning, the integration of global communication pathways, and a more formal analysis of communication-driven policy formation.

## 6. Conclusions

This paper studies the role of communication in cooperative multi-agent reinforcement learning (MARL) from the perspective of coordination under limited bandwidth. We propose a threshold-gated sparse communication framework built upon QMIX, where agents selectively activate communication based on structured changes in local observations. The communication mechanism is integrated into the utility network through neighbor-constrained aggregation, enabling communication to directly influence policy learning. Experiments on the SMAC benchmark show that the proposed approach significantly reduces communication frequency while maintaining or improving task performance. More importantly, the results reveal that, within the evaluated SMAC scenarios, communication plays a critical role in shaping policy behavior: Selective communication leads to more structured coordination patterns, including improved focus-fire consistency, more effective micro-control, and more stable training dynamics. These findings should therefore be interpreted within the specific experimental settings considered in this paper, rather than as a general claim about all cooperative MARL systems. Overall, the results suggest that, for the evaluated tasks, behavior-aware and selective communication can be more beneficial than frequent message exchange, highlighting sparse communication as a promising mechanism for regulating coordination under limited bandwidth.

## Figures and Tables

**Table 1:** Communication threshold statistics across SMAC maps. The stable-phase mean $\left(\mu_{s}\right)$ is computed from a converged reference setting without excess-activation regularization. The reported range corresponds to the minimum and maximum trigger rates observed in the stable phase.

Map	Initial Trigger Rate	Stable-Phase Mean ($\mu_{s}$)	$\delta_{\mathrm{low}}$	$\delta_{\mathrm{mid}}$	$\delta_{\mathrm{high}}$
MMM	0.510±0.068	0.308±0.016	0.308	0.358	0.408
2s3z	0.482±0.052	0.298±0.020	0.298	0.348	0.398
10m_vs_11m	0.523±0.079	0.299±0.014	0.299	0.349	0.399
1c3s5z	0.503±0.063	0.306±0.017	0.306	0.356	0.406

**Table 2:** Preliminary sensitivity analysis of the positive bias relative to $\mu_{s}$ (all values in $\times 10^{-3}$).

Bias	0.025	0.05	0.075	0.10	0.125	0.15
ally_minsep	5.50	4.61	4.31	4.04	4.31	4.11
Diff. from $\mu_{s}$ baseline	0.83	1.76	2.06	2.34	2.07	2.27

**Table 3:** Final win rate under different communication thresholds across SMAC scenarios, averaged over the last 80 evaluation records. Best results are highlighted in bold.

Map	QMIX	$\delta_{\mathrm{low}}$	$\delta_{\mathrm{mid}}$	$\delta_{\mathrm{high}}$
2s3z	0.972±0.012	0.973±0.015	0.966±0.014	0.974±0.010
MMM	0.921±0.028	0.966±0.018	0.952±0.022	0.954±0.020
10m_vs_11m	0.856±0.030	0.873±0.022	0.884±0.018	0.879±0.024
1c3s5z	0.941±0.060	0.952±0.045	0.948±0.040	0.955±0.038

**Table 4:** Summary of behavior-level coordination metrics under different communication thresholds. Values are estimated from the converged segments of Figure 5 and reported as mean ± standard deviation. Higher attack_move ratio, focus-fire, and ally minimum separation indicate stronger coordination, while lower bucket entropy indicates more concentrated target selection.

Scenario	Attack_Move Ratio	Focus-Fire	Ally Minsep	Bucket Entropy
2s3z $\_\delta_{\mathrm{low}}$	0.20±0.03	2.35±0.12	0.0546±0.0049	0.43±0.03
2s3z $\_\delta_{\mathrm{mid}}$	0.22±0.05	2.36±0.12	0.0512±0.0035	0.44±0.04
2s3z $\_\delta_{\mathrm{high}}$	0.21±0.05	2.35±0.10	0.0525±0.0020	0.44±0.03
10m_vs_11m $\_\delta_{\mathrm{low}}$	0.88±0.08	3.28±0.16	0.0072±0.0013	0.28±0.02
10m_vs_11m $\_\delta_{\mathrm{mid}}$	0.75±0.06	3.13±0.13	0.0064±0.0007	0.26±0.02
10m_vs_11m $\_\delta_{\mathrm{high}}$	0.86±0.10	3.36±0.17	0.0065±0.0008	0.27±0.02
MMM $\_\delta_{\mathrm{low}}$	0.94±0.10	3.47±0.25	0.0117±0.0020	0.31±0.03
MMM $\_\delta_{\mathrm{mid}}$	0.87±0.11	3.02±0.22	0.0132±0.0028	0.25±0.03
MMM $\_\delta_{\mathrm{high}}$	0.67±0.10	2.89±0.22	0.0117±0.0017	0.24±0.03
1c3s5z $\_\delta_{\mathrm{low}}$	0.57±0.08	2.98±0.30	0.0070±0.0024	0.34±0.04
1c3s5z $\_\delta_{\mathrm{mid}}$	0.60±0.10	2.78±0.24	0.0080±0.0017	0.36±0.04
1c3s5z $\_\delta_{\mathrm{high}}$	0.54±0.08	3.25±0.25	0.0082±0.0010	0.33±0.04

## Data Availability

The data is sourced from the SMAC. They are available on request from the authors.

## Abbreviations

The following abbreviations are used in this manuscript:

MARL	Multi-Agent Reinforcement Learning
CTDE	Centralized Training with Decentralized Execution
SMAC	StarCraft Multi-Agent Challenge
QMIX	Monotonic Q-value mixing method for value decomposition
VDN	Value-Decomposition Networks
TD	Temporal-Difference
CNN	Convolutional Neural Network
GRU	Gated Recurrent Unit
STE	Straight-Through Estimator
DQN	Deep Q-Network
MAPPO	Multi-Agent Proximal Policy Optimization
MADDPG	Multi-Agent Deep Deterministic Policy Gradient
PyMARL	Python Multi-Agent Reinforcement Learning framework
2s3z	SMAC scenario with two Stalkers and three Zealots
10m_vs_11m	SMAC scenario with ten allied Marines versus eleven enemy Marines
MMM	SMAC Marine–Marauder–Medivac scenario
1c3s5z	SMAC scenario with one Colossus, three Stalkers, and five Zealots

## Appendix A

We have provided the network configuration in the Table A1 and the time using for training in Table A2. For each task, we trained our model on a NVIDIA 4090 GPU. We provided 1.2 million to 2 million training steps for the smac environment.

**Table A1:** Experimental settings and hyperparameter configurations.

Item	Configuration
StarCraft II version	4.10, Build B75689
Maps	2s3z, 10m_vs_11m, MMM, 1c3s5z
Random seeds	3 independent seeds
Training timesteps	2.05M
Difficulty	7
Step multiplier	8
Communication radius	All allied agents
Message dimension	64
RNN hidden dimension	64
Encoder hidden dimension	128
Trigger hidden dimension	64
Attention hidden dimension	64
Learning rate	$5\times 10^{-4}$
Discount factor γ	0.99
Batch size	64
Replay buffer size	20,000
Target update interval	200
Gradient clipping norm	10
Exploration schedule	ϵ: 1.0 to 0.05 over 200,000 steps
QMIX mixing embedding dim	32
QMIX hypernetwork	2 layers, hidden dim 64
Communication weight $\lambda_{\mathrm{comm}}$	0.001
Optimizer	RMSprop, α=0.99, $\epsilon =10^{-5}$
Activation	ReLU

**Table A2:** Training time.

Task	Time (h)
MMM	25.5±0.8
10m_vs_11m	23.3±1.6
1c3s5z	23.1±1.8
2s3z	15.4±1.1
